# A phase 3 study of ravulizumab to protect patients with chronic kidney disease from cardiac surgery-associated acute kidney injury and major adverse kidney events (ARTEMIS)

**DOI:** 10.1186/s13063-025-08895-7

**Published:** 2025-05-30

**Authors:** Marlies Ostermann, David C. Corteville, Kent Doi, Jay L. Koyner, Andre Lamy, Gerry Li, Christine M. Solinsky, Pamela D. Winterberg, William T. Smith, Ravindra L. Mehta, Patrick T. Murray, Andrew D. Shaw, Alexander Zarbock, Daniel T. Engelman

**Affiliations:** 1 Critical Care and Nephrology, NHS Foundation Trust, Guy’s and St Thomas, London, UK; 2 Department of Cardiology, Rochester Regional Health, Rochester, USA; 3 Emergency and Critical Care Medicine, The University of Tokyo Hospital, Tokyo, Japan; 4 Section of Nephrology, The University of Chicago, Chicago, USA; 5 Division of Cardiac Surgery and Population Health Research Institute, McMaster University, Hamilton, Canada; 6 Alexion, AstraZeneca Rare Disease, Boston, USA; 7 Department of Medicine, San Diego School of Medicine, University of California, San Diego, USA; 8 University College Dublin Clinical Research Centre, UCD School of Medicine, Dublin, Ireland; 9 Department of Intensive Care and Resuscitation, Cleveland Clinic, Cleveland, USA; 10 Department of Anesthesiology, Intensive Care and Pain Medicine, University of Münster, Münster, Germany; 11 Heart & Vascular Program Baystate Health, University of Massachusetts Chan Medical School-Baystate, Springfield, USA; 12 Present Address: Vera Therapeutics, Brisbane, USA; 13 Present Address: Novartis, East Hanover, USA; 14 Present Address: Critical Care and ECMO Division of ABIOMED, J&J MedTech, Cleveland, USA

**Keywords:** Cardiac surgery-associated acute kidney injury, Cardiopulmonary bypass, Chronic kidney disease, Complement activation, Interventional study, Ischemia–reperfusion injury, Ravulizumab

## Abstract

**Background:**

Cardiac procedures, particularly those requiring cardiopulmonary bypass (CPB), are associated with the development of cardiac surgery-associated acute kidney injury (CSA-AKI). Development of CSA-AKI occurs as a result of inflammation, uncontrolled complement activation, and kidney cell damage. In patients with preoperative renal impairment, such as those with chronic kidney disease (CKD), there is an increased risk of both CSA-AKI and poorer clinical outcomes. Currently, there are limited effective, targeted pharmacological interventions for the prevention or treatment of CSA-AKI, although emerging therapies are being investigated, particularly in patients with existing CKD. The ARTEMIS (R*A*vulizumab to P*R*otect Pa*T*ients with Chronic Kidney Dis*E*ase fro*M* CSA-AK*I* and *S*ubsequent Major Adverse Kidney Events) trial will assess the efficacy and safety of ravulizumab (a complement C5 inhibitor) in reducing the risk of major adverse kidney events (MAKE) in patients with preoperative CKD undergoing non-emergent cardiac surgery with CPB.

**Methods:**

This trial is currently recruiting patients with CKD who have planned cardiac surgery requiring CPB including coronary artery bypass grafting, valve replacement or repair, or combined procedures. This is a phase 3, randomized, double-blind, placebo-controlled, global study assessing the efficacy and safety of a single preoperative dose of ravulizumab. These outcomes will be assessed using the occurrence of MAKE and its components, as well as the occurrence and severity of CSA-AKI throughout the study period.

**Discussion:**

Complement activation is known to occur during and after cardiac procedures as a result of CPB and ischemia–reperfusion injury, leading to a cycle of cell damage and death. Therefore, it is hypothesized that preoperative administration of ravulizumab will provide immediate and complete complement inhibition, which will be sustained throughout the surgical period, preventing the uncontrolled complement activation associated with the development of CSA-AKI, thus minimizing poor outcomes for patients.

**Trial registration:**

ClinicalTrials.gov NCT05746559. Registered on February 27, 2023.

**Supplementary Information:**

The online version contains supplementary material available at 10.1186/s13063-025-08895-7.

## Background

Cardiac surgery-associated acute kidney injury (CSA-AKI) is a common postoperative complication, particularly following procedures conducted on cardiopulmonary bypass (CPB). Development of acute kidney injury (AKI) is associated with substantial morbidity—including increased infection risk, prolonged mechanical ventilation, development/progression of chronic kidney disease (CKD), need for kidney replacement therapy (KRT), and higher rates of hospital readmission—alongside increased mortality [1, 2]. The composite endpoint of major adverse kidney events (MAKE) is comprised of the following variables: mortality, requirement for KRT, and sustained kidney dysfunction (≥ 25% decline from baseline in estimated glomerular filtration rate (eGFR)). Clinical trials investigating AKI routinely include MAKE as a key efficacy outcome, as the components are easily quantifiable indicators of kidney function [3, 4]. Patients with CKD are at a substantially increased risk of developing CSA-AKI (> 50% vs ~ 25%) and MAKE, leading to higher rates of long-term mortality (1–5 years post-surgery) compared with patients without prior CKD [2, 5–7]. Currently, there are limited effective, targeted pharmacological interventions for the prevention or treatment of CSA-AKI, and several therapies have failed to demonstrate efficacy in clinical studies [2, 8–10]. A recently completed phase 3 trial of amino acid infusion prior to cardiac surgery demonstrated positive efficacy (particularly in patients with CKD); however, the effectiveness of this approach remains to be fully elucidated and validated in real-world clinical practice [11–14]. Further trials are planned for other therapeutic approaches [15, 16].

In addition to the general risks associated with surgery, kidney injury may occur due to inflammation and hypoperfusion triggered by CPB [17]. Kidney hypoxia can cause injury to and death of both tubular epithelial cells and kidney endothelial cells, while further injury may also occur upon reperfusion. This process, termed ischemia–reperfusion injury (IRI), is a key driver of CSA-AKI pathogenesis [17, 18]. Additionally, direct contact between the blood and CPB circuitry, alongside shear stress on red blood cells whilst on bypass, are both known to cause hemolysis [19, 20]. This results in the generation of free heme which induces oxidative stress and, in addition to subsequent damage to kidney cells, causes the release of proinflammatory mediators, damage-associated molecular patterns, and direct activation of the complement system [20–22]. Complement activation results in the formation of the proinflammatory anaphylatoxins C3a and C5a, and the cytolytic membrane attack complex (MAC, C5b-9) [23]. These components of the terminal complement cascade cause further damage to kidney cells, perpetuating and amplifying a cycle of inflammation and injury [24, 25].

Ravulizumab, a humanized monoclonal antibody, binds complement C5 and provides immediate, complete, and sustained inhibition of the terminal complement pathway [26, 27]. Ravulizumab has a well-established safety and tolerability profile and is already approved for use in several complement-mediated disorders [26, 28–30]. Preventing injury to the kidney by inhibiting terminal complement may result in a decrease in the incidence, severity, and duration of CSA-AKI and reduce consequent MAKE. Proof of concept for this efficacy effect is derived from published reports of the effectiveness of C5 inhibition in patients with aHUS undergoing kidney transplantation, patients undergoing coronary artery bypass grafting with CPB, and higher-risk cardiac surgery patients [31–34].

Here we describe the study design for ARTEMIS (R*A*vulizumab to P*R*otect Pa*T*ients with Chronic Kidney Dis*E*ase fro*M* CSA-AK*I* and *S*ubsequent Major Adverse Kidney Events) which will assess the efficacy and safety of ravulizumab in reducing the risk of MAKE in patients with preoperative CKD undergoing non-emergent cardiac surgery with CPB (NCT05746559).

## Methods

**Fig. 1:**
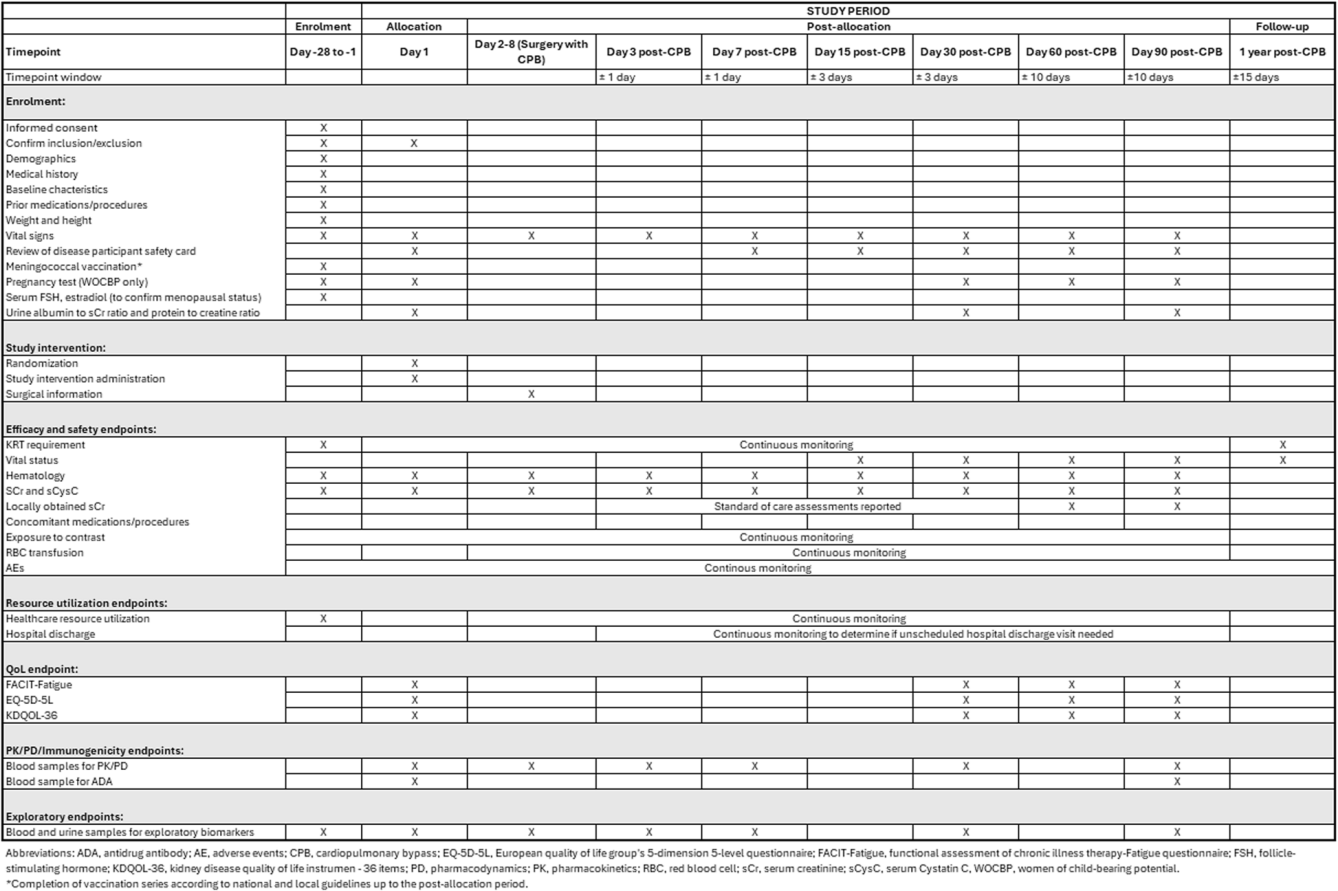
SPIRIT Figure study period schedule

SPIRIT guidelines were followed in the reporting of this trial [35]. The completed SPIRIT checklist can be found in Additional File 1, and the SPIRIT Figure detailing the full study schedule is included as Fig. 1.

### Study design

ARTEMIS is a phase 3, randomized, double-blind, placebo-controlled, global study of ravulizumab in adult participants with CKD undergoing non-emergent cardiac surgery requiring CPB for coronary artery bypass graft (CABG), valve replacement or repair, or combined procedures. The primary objective of this study is to assess the efficacy of ravulizumab in reducing the risk of MAKE at 90 days following surgery with CPB (MAKE90).

Participants are included on the basis of having preoperative CKD and considered at risk for further kidney events after CPB due to preoperative eGFR values of ≥ 20 to < 60 mL/min/1.73 m^2^ and a minimum Society of Thoracic Surgeons (STS) Calculator Renal Failure Risk Score of ≥ 2.8%; the STS score will be calculated during the initial screening period. The STS Calculator is a model which utilizes patient characteristics and baseline clinical data to generate procedure-specific risk scores for patient outcomes [36, 37]. Participants are identified by qualified research staff via methods including review of medical records, external referrals, or use of databases. Recruitment strategies may include study posters, referral letters, recruitment brochures, advertisements, social media posts, and websites, where permitted by local regulations. All recruitment materials will be submitted to local institutional review boards/ethics committees for review and approval, as required, prior to use. This will be a global study conducted in at least 19 countries.

**Fig. 2:**
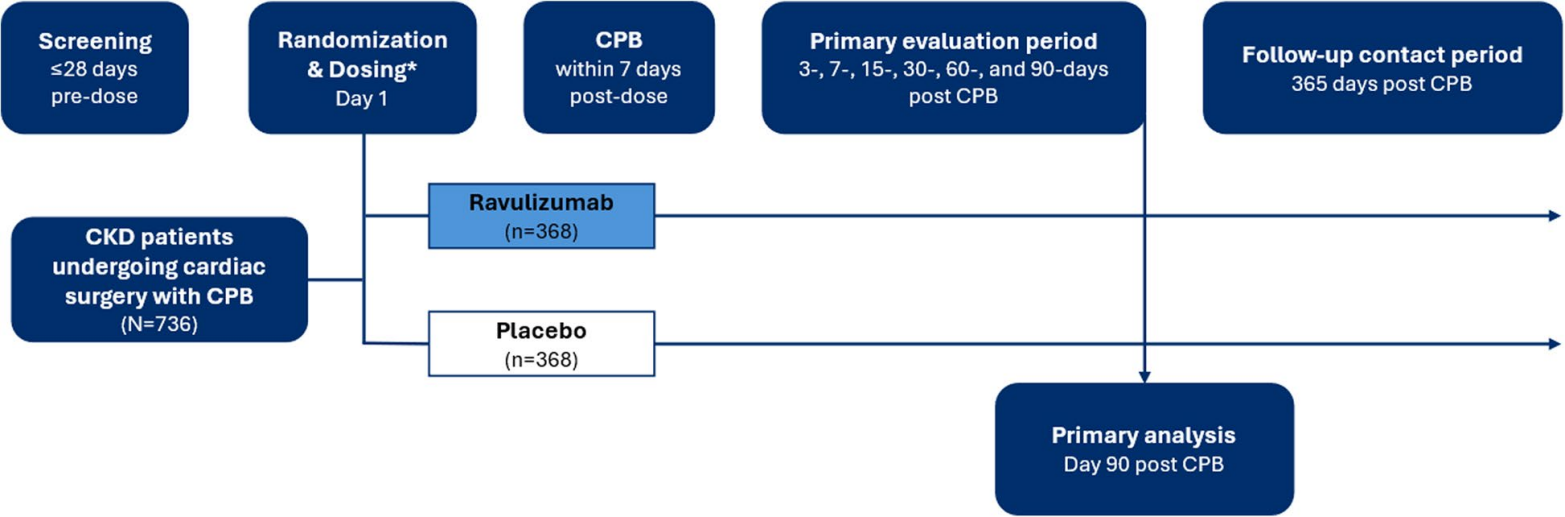
ARTEMIS study design. *Weight-based dosing will ensure ravulizumab serum concentrations of ≥ 175 μg/mL and achieve complete terminal complement inhibition, while maintaining maximum ravulizumab serum concentrations below the highest observed value of 3000 μg/mL from all completed clinical studies, for ≥ 18 days in the target population

Following the initial 28-day screening period, randomization and dosing will occur within 7 days prior to surgery with CPB (Fig. 2). Participants will be assessed on Days 3, 7, 15, 30, 60, and 90 after surgery (primary evaluation period), with the primary endpoint analysis conducted at Day 90 post CPB (primary analysis). Serum creatinine (sCr) and serum cystatin C (sCysC) will be measured on the day of screening (baseline); day of dosing (day 1); day of surgery (prior to induction of anesthesia), daily from days 1 to 7; on days 15, 30, 60, and 90 post CPB; and at hospital discharge. Baseline eGFR is reported as the average eGFR calculated from the last assessment during the screening and day 1 visits, prior to study intervention administration. Participants will be contacted by telephone at one year post CPB to assess the requirement for KRT and survival (survival follow-up period). The total study duration is approximately 400 days. A participant will be considered to have completed the study once they have completed the primary evaluation period or withdrawn.

A global, multidisciplinary executive steering committee (ESC) of independent experts in the field was established in 2023. Since its inception, the ESC meets every 6 months, providing input into the ravulizumab CSA-AKI global clinical development program and acting in an advisory capacity to Alexion on the study. The study started in April 2023, and the current estimated completion date is February 2027.

### Study endpoints

The primary endpoint in this study is the occurrence of MAKE90, defined as meeting at least one of the following events post CPB: initiation of KRT or death from any cause through day 90, or a decrease from baseline eGFR of ≥ 25% at day 90 calculated using the CKD-EPI equation and sCysC. This endpoint was chosen following discussions with and recommendations from both the Food and Drug Administration (FDA) and European Medicines Agency (EMA). The primary estimand is the population-level treatment difference between the two treatment groups in the proportion of participants experiencing MAKE90. The primary analysis will be based on the intent-to-treat (ITT) analysis set, which consists of all participants randomized to study intervention, under the treatment policy strategy. In this strategy, the values of the outcome of interest are used regardless of the occurrence of any intercurrent events. The treatment difference will be estimated using the Cochran-Mantel Haenszel (CMH) method adjusting for the two stratification variables (surgery type and baseline CKD stage). For the primary endpoint, missing data for KRT initiation or death will be imputed as a non-event, and missing eGFR values will be handled by multiple imputation in two steps. First, data will be imputed using Markov chain Monte Carlo to create a monotonic missing data pattern; then the second step will use the regression method for monotone missing data to impute the remaining missing observations. Rubin’s rule will be used for combining results to yield multiple imputation point estimates and standard errors. To minimize the risk of missing data, Alexion have a variety of patient retention initiatives including, but not limited to: home health and telehealth options for patients located substantial distances from clinical centers; clear and frequent communications provided to sites on the importance of complete data collection; active engagement with sites where patients are lost to follow-up to improve retention; and active site monitoring throughout the study. This allows for any issues to be addressed as they arise and prevents the development of more substantial complications.

Analysis of key secondary endpoints will also be based on the ITT analysis set and will include the occurrence and severity of Kidney Disease: Improving Global Outcomes (KDIGO)-defined AKI; length of post-operative intensive care unit stays; occurrence of KRT or mortality; and all-cause mortality. The primary assessment of MAKE90 will be based on sCysC measurements, as it is known that skeletal muscle atrophy (a common complication of cardiac surgery) can decrease sCr levels, leading to overestimation of eGFR after cardiac surgery [38, 39]. The secondary endpoints measuring AKI occurrence will be based on the sCr measurement. Additional secondary endpoints include MAKE assessed at days 30 and 60 post CPB using eGFR calculated from sCr or sCysC, MAKE assessed at day 90 post CPB using eGFR calculated from sCr, and occurrence of STS renal failure as determined by the Risk, Injury, Failure, Loss of kidney function, and End-stage kidney disease (RIFLE) Failure criteria. The proportion of participants meeting each of the key secondary endpoints will be summarized by treatment group. The point estimates and associated 2-sided 95% confidence intervals of the treatment difference in proportions will be estimated using the CMH method. Healthcare resource utilization will be assessed by documenting and comparing the length of total index hospital stays, number of days on mechanical ventilation, hospital readmissions, and days on KRT between treatment groups. Safety, pharmacokinetics and pharmacodynamics, and patient-reported outcomes of health-related quality of life will also be assessed. Exploratory endpoints in this study include exploration of the efficacy of ravulizumab in reducing the risk of CSA-AKI within 7 days of CPB, measured via sCysC (highest AKI stage by KDIGO criteria and/or occurrence of severe AKI [KDIGO stage 2 or 3]), and the assessment of biomarkers at baseline and following treatment. Biomarkers include, but are not limited to: complement pathway activation (plasma and urine sC5b-9), renal injury (urine neutrophil gelatinase-associated lipocalin), and endothelial damage (plasma thrombomodulin).

A series of subgroup analyses of the primary endpoint and key secondary endpoints are planned, including in the following participants: different races; different sexes; different age groups (18–60, 61–75, > 75 years); different CKD stages at baseline; and different surgical types during CPB.

### Study assessments and procedures

Study inclusion and exclusion criteria are listed in Table 1. The minimum Renal Failure Risk Score will additionally ensure that participants who are at risk for postoperative kidney events are enrolled [36, 37]. Baseline eGFR (and subsequent measures on days 30, 60, and 90) will be determined using the CKD Epidemiology Collaboration (CKD-EPI) equation based on central measurements of sCysC and/or sCr, as described above.

**Table 1:** Description of the key inclusion and exclusion criteria for enrollment in this study

Inclusion criteria	Exclusion criteria
≥ 18– ≤ 90 years of age at the time of informed consent	If expected or planned surgery is: - Emergency or salvage (expected at Screening or Randomization, as assessed by the investigator) - Single-vessel CABG without valve surgery - Surgery conducted without CPB (off-pump)
Body weight ≥ 30 kg at screening period	Any use of KRT or presence of AKI within 30 days prior to randomization (except transient, ≤ 5 days, stage 1 AKI after iodinated contrast exposure); the presence of AKI must be assessed within 72 h prior to randomization
Planned non-emergent cardiac surgery requiring CPB, for these procedures: - Multi-vessel CABG - Valve replacement or repair (ascending aorta surgery permitted if combined with aortic valve replacement/repair) - Combined CABG and valve surgery (inclusion of single-vessel CABG is permitted when combined with valve replacement/repair)	Cardiogenic shock or hemodynamic instability within 72 h prior to randomization (including use of intra-aortic balloon pump or other temporary cardiac output support device, extracorporeal membrane oxygenation, or left ventricular assist device)
Surgery scheduled to occur within 35 days of screening visit (to allow 28 days screening with dosing 1 to 7 days prior to CPB)	Solid organ or bone marrow transplant recipient
Known or apparent CKD (evaluated by history, diagnostic results, or reasonable assessment by the investigator) and eGFR ≥ 20– < 60 mL/min/1.73 m^2^ calculated using the CKD-EPI equation by sCr or sCysC levels, during screening period	History of infection including: - Active systemic bacterial, viral, or fungal infection within 14 days prior to randomization - A history of unexplained, recurrent infection - History of, or unresolved *N. meningitidis* infection
Minimum STS Calculator Renal Failure Risk Score of ≥ 2.8% during screening period	History of human immunodeficiency virus infection who are not on anti-retroviral therapy or if on therapy have a known detectable viral load within 1 year prior to screening
Capable of giving informed consent	Congenital immunodeficiency
	Known medical or psychological condition(s), including substance abuse, or risk factor that might interfere with the participant’s full participation in the study, pose any additional risk for the participant, or confound the assessment of the participant or outcome of the study
	Hypersensitivity to any ingredient in the study interventions, including murine proteins
	Current malignancy (excluding local or regional prostate cancer or non-melanoma skin cancer, or indolent disease not being treated) or receiving treatment for malignancy
	Prior or concomitant therapy including: - Complement inhibitors, or plasmapheresis or plasma exchange within the year prior to screening or during study - Use of IVIg (e.g., as acute therapy) within 4 weeks prior to dosing - Planned use of any agent specifically for prevention of AKI
	Anticipated use of: - KRT - Extracorporeal membrane oxygenation, or temporary cardiac support devices between randomization and surgery*
	Participation in another interventional treatment study or use of any experimental therapy within 30 days before day 1 in this study, or within five half-lives of that investigational product
	Presence of a do-not-resuscitate order or life expectancy of < 3 months
	Pregnant, breastfeeding, or intending to conceive within 8 months after the dosing period
	Participant is unwilling to be vaccinated against *N. meningitidis* or unwilling to receive prophylactic antibiotic treatment if needed

* Elective or preemptive insertion of temporary cardiac support devices (including use of an intra-aortic balloon pump) on the day of surgery is acceptable in the absence of cardiogenic shock or hemodynamic instability

Approximately 736 participants will be randomly assigned (1:1) using Interactive Response Technology (IRT) and the block randomization method to receive either ravulizumab or placebo, stratified by baseline CKD stage (3a, 3b, 4) and surgery type (mitral valve replacement or combined procedures vs other single procedure). The sample size estimation was based on a two-proportion difference using a normal approximation method to compare the treatment difference in the proportion of participants experiencing MAKE events within 90 days post CPB between the ravulizumab and the placebo group. A sample size of 736 (368 participants per treatment group) has at least 90% power to detect a statistically significant treatment difference of 10% in the proportion of participants with MAKE within 90 days post CPB under a 2-sided significance level of 0.05.

Participants, all investigative site personnel, and any Alexion employee or designee directly associated with the conduct of the study will be blinded to participant treatment assignment throughout the study. The blinding will be maintained by using identical study intervention kits, labels, and appearance of ravulizumab and placebo. The randomization code will be maintained by the IRT provider. In case of an emergency, the investigator has the sole responsibility for determining if unblinding of a participant’s intervention assignment is warranted, primarily based on participant safety. If the investigator decides that unblinding is warranted, the investigator may, at the investigator’s discretion, contact Alexion to discuss the situation prior to unblinding a participant’s intervention assignment unless this could delay emergency treatment for the participant. If a participant’s intervention assignment is unblinded, Alexion must be notified within 24 h after breaking the blind. The date and reason that the blind was broken must be recorded in the source documentation and case report form (CRF), as applicable.

In the case of unblinding, unblinded information will only be accessible to those who are involved in the safety reporting to Health Authorities, Independent Ethics Committees (IECs), Institutional Review Boards (IRBs), and/or the independent data monitoring committee (DMC). When unblinding is the result of an AE that is unexpected or related and serious, the blind will be broken for that specific participant only. In the case of unblinding (for example, due to safety concerns), persons responsible for ongoing study conduct and subsequent data analysis and interpretation will remain blinded.

Randomization should occur on the same day as dosing, except in cases when the study intervention needs to be prepared the day before dosing. Screening and randomization or randomization and surgery may occur on the same calendar day. Participants will receive a single weight-based intravenous dose of either placebo or ravulizumab on day 1 (Table 2) and surgery must be scheduled to occur within 7 days after dosing. In the event of unexpected surgical delay, surgery with CPB should occur no later than 15 days after study drug infusion. Study drug infusion must be completed at least 1 h prior to the surgery start. This schedule is based on predictions by model-based simulations that ravulizumab will provide complete inhibition of C5 for at least 18 days. These simulations combined data on serum concentrations of ravulizumab over time in patients with atypical hemolytic uremic syndrome [29] with data from two studies (NCT04543591 and NCT04557735) on the effect of blood transfusion on ravulizumab clearance in patients with hematopoietic stem cell transplantation–associated thrombotic microangiopathy.

**Table 2:** Weight-based single dose of ravulizumab or placebo given within 7 days prior to cardiac surgery with CPB

Body weight (kg)	Dose (mg)
≥ 30– < 40	2700
≥ 40– < 60	3000
≥ 60– < 100	3300
≥ 100	3600

Each 30-mL single-use vial of ravulizumab contains 300 mg of ravulizumab (10 mg/mL), 10 mM sodium phosphate, 150 mM sodium chloride, and 0.02% polysorbate 80 in water at pH 7.0. The placebo is formulated as a matching sterile solution with the same buffer components, without ravulizumab. All participants will receive investigator-determined standard of care in addition to the study interventions (according to institutional practices and participant characteristics) unless these are listed as prohibited concomitant therapies within the exclusion criteria (Table 1).

### Data collection and management

All participant study data will be recorded on the case report form (CRF) unless transmitted to Alexion or designee electronically. The investigator is responsible for verifying that data entries are accurate and correct by maintaining source data that supports the information in the CRF. The investigator must permit study-related monitoring, audits, IRBs/IECs review, and regulatory agency inspections, and provide direct access to source data documents. Alexion or designee is responsible for the data management of this study, including quality checking of the data. Records and documents, including signed informed consent forms, pertaining to the conduct of this study must be retained by the investigator for at least 25 years after study completion, or longer if required by local regulations or institutional policies. No records may be destroyed or transferred to another location/party without the written approval of Alexion. Clinical study documents and records required as part of the trial master file are archived and stored by Alexion for at least 30 years. Quality tolerance limits will be predefined to identify systematic issues that can impact participant safety and/or reliability of study results. These predefined parameters will be monitored during the study, and important deviations and remedial actions taken will be summarized in the clinical study report.

### Monitoring of harms

All adverse events (AE)s, including administration-related AEs, will be reported to the investigator or qualified designee by the participant (or surrogate when appropriate). The investigator is responsible for recording AEs or serious AEs (SAE) and remains responsible for following up on SAEs that are serious, considered related to the study, or cause the participant to discontinue. All AEs will be collected from the signing of the informed consent form until the end of the primary evaluation period; all SAEs will be recorded and reported to Alexion or the designee immediately, and under no circumstances should this exceed 24 h. Any updated SAE data will be submitted to Alexion or the designee within 24 h of becoming available.

Any dose of study intervention greater than that specified in the protocol will be considered an overdose. Accidental or suspected overdose without any association with laboratory abnormalities or clinical symptoms should not be considered an AE unless there are negative clinical consequences, but must be reported by the investigator to Alexion. No specific treatment for overdose is recommended.

Treatment-emergent AEs (TEAEs) are defined as AEs with onset (or existing events that worsen in severity) from the initiation of dosing of study intervention through 90 days post CPB. All AEs will be coded using the Medical Dictionary for Regulatory Activities, version 24.1 or higher, and will be summarized by System Organ Class and Preferred Term overall, by severity, and by relationship to study intervention. Detailed by-participant listings of all TEAEs (including those leading to withdrawal from the study) will be reported. Participants who experience a reaction during the administration of ravulizumab should be treated according to institutional guidelines. Alexion has insurance to cover the costs of research injuries as long as the participant followed the investigator’s instructions, the costs are reasonable, and the participant did not cause the injury.

Patients receiving complement inhibitors are at increased risk of infection by *N. meningitidis*. All participants in ARTEMIS must be vaccinated against *N. meningitidis* within the 3 years prior to enrollment or during the screening period; hospitalized participants may be vaccinated after study intervention administration but prior to hospital discharge. Vaccines against serotypes A, C, Y, and W135 (and serotype B where available if recommended by local guidelines) are recommended in preventing commonly pathogenic meningococcal serotypes. Participants must receive vaccination series as indicated according to current national vaccination guidelines. If ravulizumab dosing occurs less than 2 weeks after initial vaccination, participants will receive appropriate prophylactic antibiotics for at least 2 weeks after vaccination. All participants will also be monitored for early signs of meningococcal infection and treated if required. Furthermore, prior to study intervention administration, a safety card will be provided to participants to increase participant awareness of the risk of meningococcal infection, promote quick recognition of the signs/symptoms of infection, and inform participants on what actions must be taken if they are experiencing these.

Intravenous and infusion-related reactions are a potential risk with the use of monoclonal antibodies; these reactions can be nonimmune or immune mediated (e.g., hypersensitivity reactions). All administration, intravenous, and infusion-related reactions will be reported to the Investigator and qualified designee. The Investigator and qualified designee are responsible for detecting, documenting, and recording events that meet the definition of AE or SAE and remain responsible for following up on events that are serious, considered related to the study intervention, or study procedures; or that caused the participant to discontinue ravulizumab.

The primary analysis will occur when all enrolled participants have completed the primary evaluation period. An independent DMC, comprising experts in relevant fields with no direct relationship to the study, has been appointed by Alexion to assess safety and efficacy measures. The DMC will review unblinded study information provided by an independent data analysis center during the conduct of the study for periodic safety review (at least quarterly) and two planned interim analyses. The two interim analyses are planned after approximately 30% and 50% of the randomized participants have completed the primary evaluation period (i.e., day 90 post CPB Visit). At each interim analysis, the DMC will make formal recommendations based on the pre-specified criterion contained in the DMC statistical analysis plan. The purpose of the first interim analysis is to assess early futility, while the second interim analysis will include futility, early efficacy, and sample size re-estimation assessments. Any amendments to the protocol will be submitted for IRB/IEC and health authority approval before the implementation of changes made to the study design, except for changes necessary to eliminate an immediate hazard to study participants. All amendments to date will be reported in the amended protocol document, which will be provided to all study sites/investigators as well as updated on all relevant clinical trial registries. Following any protocol amendments, specific training will be conducted with sites to ensure compliance. When a protocol deviation is identified—for example, during the active monitoring of sites conducted by Alexion and its appointees—a thorough deviation standard operating procedure (SOP) is in place to investigate, classify, report, and mitigate the identified issue(s).

## Discussion

Patients with CKD undergoing cardiac surgeries with CPB have both a greater risk of developing CSA-AKI and poorer outcomes than those without CKD, with no targeted therapies currently available to prevent or reduce these risks and improve patient outcomes [5, 7]. Here, we describe the study design and rationale for ARTEMIS, an ongoing pivotal phase 3 trial aiming to assess the efficacy and safety of preventive terminal complement inhibition to improve outcomes in patients with CKD undergoing cardiac surgery with CPB. The primary endpoint in this study is the occurrence of the composite outcome MAKE. This has historically been recommended for assessing efficacy in AKI clinical trials [3, 4] to allow comparisons with similar clinical trials. However, it is of note that there can be heterogeneity in the components assessed between trials and this should be considered when comparisons are being made [40]. A preoperative dose of ravulizumab will provide immediate, complete, and sustained inhibition of terminal complement activity throughout CPB, IRI, and during the second wave of complement activation seen 2–3 days post-surgery [24, 41–43]. Additionally, the long-term C5 inhibition provided by ravulizumab provides logistical flexibility and permits outpatient preoperative administration.

Patients receiving complement inhibitors are at a 1000–2000-fold increased risk of *N. meningitidis* infection compared to the general population [44]. However, previous studies have demonstrated that this is well managed in both clinical and real-world settings [45, 46]. The favorable tolerability of ravulizumab and implementation of strategies to mitigate the risk of infection support its use in this study.

Complement inhibition in patients with CKD undergoing cardiac surgery with CPB may be beneficial in improving outcomes and prognoses by minimizing AKI and MAKE risks, including mortality. The ARTEMIS trial aims to establish the safety and efficacy of terminal complement inhibition with ravulizumab in preventing or reducing the deleterious effects of kidney injury following cardiac surgery in participants with preexisting CKD.

## Supplementary Information

Supplementary Material 1. SPIRIT checklist.

Supplementary Material 2.

## Data Availability

Alexion, AstraZeneca Rare Disease will consider requests for disclosure of clinical study participant-level data provided that participant privacy is assured through methods such as data de-identification, pseudonymization, or anonymization (as required by applicable law), and if such disclosure was included in the relevant study informed consent form or similar documentation. Qualified academic investigators may request participant-level clinical data and supporting documents (statistical analysis plan and protocol) pertaining to Alexion-sponsored studies. Further details regarding data availability and instructions for requesting information are available in the Alexion Clinical Trials Disclosure and Transparency Policy. Data request form can be found here.

## Abbreviations

AE: Adverse event
AKI: Acute kidney injury
ARTEMIS: R*A*vulizumab to P*R*otect Pa*T*ients with Chronic Kidney Dis*E*ase fro*M* CSA-AK*I* and *S*ubsequent MAKE
CABG: Coronary artery bypass graft
CMH: Cochran-Mantel Haenszel
CKD: Chronic kidney disease
CKD-EPI: CKD Epidemiology Collaboration
CPB: Cardiopulmonary bypass
CRF: Case report form
CSA-AKI: Cardiac surgery associated acute kidney injury
DMC: Data monitoring committee
Egfr: Estimated glomerular filtration rate
IRI: Ischemia-reperfusion injury
IEC: Independent Ethics Committees
IRB: Institutional Review Board
IRT: Interactive response technology
ITT: Intention to treat
KDIGO: Kidney disease improving global outcomes
KRT: Kidney replacement therapy
MAC: Membrane attack complex
MAKE: Major adverse kidney events
MAKE90: Major adverse kidney events within 90 days of surgery
RIFLE: Risk, Injury, Failure, Loss of function, End-stage kidney disease criteria
SAE: Serious adverse events
sCr: Serum creatinine
sCysC: Serum cystatin C
STS: Society of Thoracic Surgeons
TEAE: Treatment-emergent adverse event
