# Supplementary material for: A phase 3 study of ravulizumab to protect patients with chronic kidney disease from cardiac surgery-associated acute kidney injury and major adverse kidney events (ARTEMIS)
Source: Trials. 2025 May 30;26:181. doi: 10.1186/s13063-025-08895-7 (PMC12125780; doi:10.1186/s13063-025-08895-7)
Supplement: Supplementary file 2 — Supplementary Material 2. [file 13063_2025_8895_MOESM2_ESM.pdf]

A Phase-3 Study of Ravulizumab to Protect Patients with Chronic Kidney Disease from Cardiac Surgery-Associated Acute Kidney Injury and Major Adverse Kidney Events (ARTEMIS)

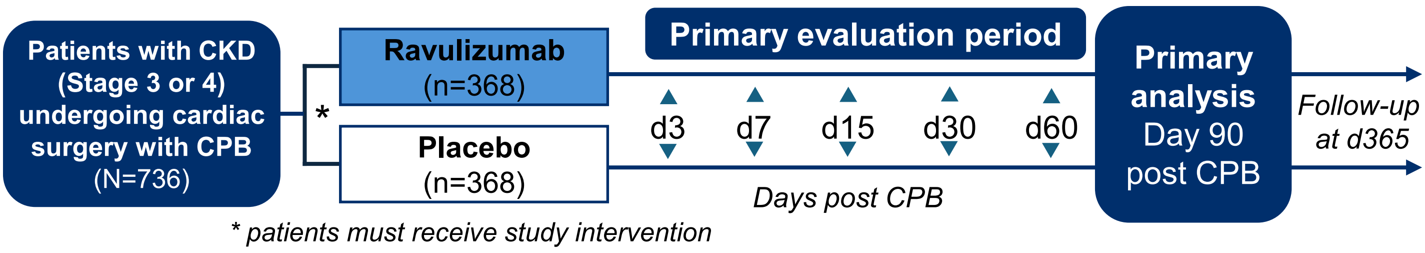

* patients must receive study intervention as a single dose 0 to 7 days prior to cardiac surgery with CPB

- Phase 3, randomized, double-blind, placebo-controlled, global study of ravulizumab in reducing the risk of clinical consequences of AKI in adults with CKD undergoing cardiac surgery with CPB
- Ravulizumab is a humanized monoclonal antibody that binds complement component 5 – providing immediate, complete, and sustained inhibition of the terminal complement pathway – preventing the formation of pro-inflammatory components
- Patients must have stage 3A, 3B or 4 CKD and have planned cardiac surgery with CPB
- Patients receive a single weight-based dose of ravulizumab or placebo 0–7 days prior to cardiac surgery with CPB
- Primary analysis occurs at 90 days post cardiac surgery with CPB, with follow-up at 365 days post surgery

Participants must be vaccinated against *N. meningitidis*

If dosing occurs < 14 days after initial vaccination, prophylactic antibiotics beginning on day of study drug infusion through 14 days after vaccination will be administered

Primary objective: Assess the efficacy of ravulizumab in reducing the risk of Major Adverse Kidney Events through day 90 post CPB (MAKE90).

***MAKE90**: at least one of the following*

Initiation of KRT by day 90

≥25% sustained decrease from baseline eGFR at day 90

Death from any cause by day 90

Key secondary endpoints:

- Occurrence and severity of KDIGO-defined AKI
- Length of post-operative intensive care unit stay
- Occurrence of KRT or mortality
- All-cause mortality

Exploratory endpoints:

- Risk of CSA-AKI within 7 days of CPB
- Assessment of biomarkers (including complement and injury markers)

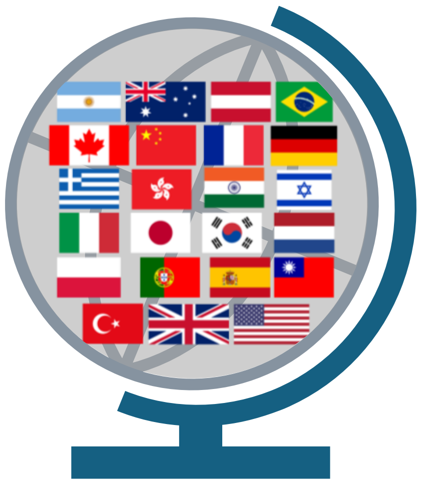

This study is recruiting (May 2025)

Currently **214** study locations over **23** countries

Abbreviations: AKI, acute kidney injury; CKD, chronic kidney disease; CPB, cardiopulmonary bypass; CSA-AKI, cardiac surgery-associated acute kidney injury; eGFR, estimated glomerular filtration rate; KDIGO, Kidney Disease: Improving Global Outcomes; KRT, kidney replacement therapy.

Preoperative administration of ravulizumab may provide immediate and complete complement inhibition which will be sustained throughout the surgical period, preventing the uncontrolled complement activation associated with development of CSA-AKI.

Ostermann, M., Corteville, D.C., Doi, K. *et al. Trials* **26**, 181 (2025).
10.1186/s13063-025-08895-7
